# Functional dependency in the direct post-ICU phase in patients with prolonged mechanical ventilation

**DOI:** 10.1186/cc11137

**Published:** 2012-03-20

**Authors:** S Vossenberg, I Drogt, N Bruins, C De Jager, EC Boerma, M Tijkotte

**Affiliations:** 1 Zorggroep Noorderbreedte, Leeuwarden, the Netherlands; 2 Medical Centre Leeuwarden, the Netherlands

## Introduction

Prolonged mechanical ventilation and length of stay (LOS) in the ICU is associated with long-term impaired functional capacity. However, little is known about functional dependency in the direct post-ICU phase. Therefore the timing and location for optimal post-ICU rehabilitation programs remain to be established.

## Methods

In this single-centre observational study we aimed to quantify functional dependency at three different time points: discharge from ICU (DI), discharge from hospital (DH) and discharge from nursing home rehabilitation unit (DR). To this end we retrospectively assed Barthel scores (BS) for individual patients [1], with a duration of mechanical ventilation >48 hours. Data are presented as median (IQR). Comparison between time points was performed with nonparametric tests for paired data and repeated measurements. *P *< 0.05 was considered significant.

## Results

Thirty-four patients were included. Baseline characteristics: APACHE II score 20 (17 to 25), age 68 (55 to 73) years, LOS ICU 22 (8 to 36) days, mechanical ventilation 8 (2 to 17) days, LOS hospital 21 (14 to 30) days, LOS rehabilitation unit 53 (31 to 85) days. Median BS at DI was 2 (1 to 3), indicating total functional dependency. In comparison to baseline, BS increased to 8 (2 to 12) at DH (*P *< 0.001), indicating severe dependency, and finally to 16 (11 to 18) at DR, indicating independency with some disabilities (*P *< 0.001). The absolute increase in BS was significantly greater during the stay in the rehabilitation unit, as compared to the general hospital ward (*P *< 0.001).

## Conclusion

ICU patients with prolonged mechanical ventilation remain severely functionally dependent after ICU discharge, but dependency reduces significantly during rehabilitation in hospital and in a nursing home rehabilitation unit.
